# Establishment of a stable monoculture system for *Entodinium furca monolobum* and isolation of *Escherichia* spp. as growth-promoting bacteria

**DOI:** 10.3389/fmicb.2026.1741192

**Published:** 2026-02-12

**Authors:** Yujia Wang, Congjie Hua, Yang Xiao, Jinying He, Bingqi Chen, Beibei Li, Dongsong Li, Tingyu Zheng, Kai Liu, Jie Xiong, Wei Miao, Jinmei Feng

**Affiliations:** 1 Department of Pathogenic Biology, School of Medicine, Jianghan University, Wuhan, China; 2 Institute of Hydrobiology, Chinese Academy of Sciences, Wuhan, China; 3 Key Laboratory of Breeding Biotechnology and Sustainable Aquaculture, Chinese Academy of Sciences, Wuhan, China; 4 Hubei Key Laboratory of Cognitive and Affective Disorders, Jianghan University, Wuhan, China; 5 Hubei Provincial Demonstration Center for Experimental Medicine Education, School of Medicine, Jianghan University, Wuhan, China

**Keywords:** *Entodinium furca monolobum*, growth-promoting bacteria, *in vitro* monoculture, protozoan–bacterial interactions, rumen ciliates

## Abstract

**Introduction:**

*Entodinium furca monolobum* is a common rumen ciliate protozoan, while it remains poorly characterized due to the lack of a stable monoculture system.

**Methods:**

In the present study, we established a stable *in vitro* monoculture of *Ent. furca monolobum* using a modified SP medium with inoculum derived from Holstein cows through filtration-based enrichment and serial dilution. We isolated bacteria from the monoculture supernatant and ciliate-associated fractions, and evaluated candidate isolates in co-culture assays. We also profiled bacterial communities by amplicon sequencing.

**Results:**

A stable *in vitro* monoculture of *Ent. furca monolobum* was established. Eight bacterial species were isolated from the monoculture supernatant and four from the ciliate-associated community. *Escherichia coli and Escherichia fergusonii* were consistently detected in both fractions and significantly promoted ciliate proliferation, with *E. coli* showing a stronger effect (*p* < 0.05). Community profiling showed distinct bacterial structures between monoculture supernatant and protozoa-free rumen fluid, and Gammaproteobacteria dominated in *E. coli*-supplemented cultures.

**Discussion:**

This work not only overcomes the long-standing challenge of maintaining *Ent. furca monolobumin vitro* culture, but also provides a methodological framework for developing defined monoxenic systems. The stable monoculture system and identification of growth-promoting *Escherichia spp.* provide a methodological basis for mechanistic studies of protozoan–bacterial interactions in the rumen.

## 1 Introduction

The rumen is a complex anaerobic microbial ecosystem. Within it, bacteria, archaea, protozoa, fungi, and other microorganisms ferment dietary carbohydrates to provide nutrients for the host (Newbold and Ramos-Morales, 2020). Rumen ciliates are the predominant protozoa in rumen, with population densities reaching 10^8^–10^9^ cells/L of rumen fluid in domestic ruminants, and accounting for up to 50% of the total microbial biomass (Shin et al., 2004; Newbold et al., 2015). They play vital roles in feed digestion and fermentation, influence methanogenesis, modulate nitrogen utilization efficiency, and contribute to the stability of the ruminal microbial community through complex interactions with other microorganisms (Bonhomme, 1990; Williams and Coleman, 1992; Newbold et al., 2015; Firkins et al., 2020).

Rumen ciliates are morphological diversity and belonging to the orders Vestibuliferida and Entodiniomorphida (Lynn, 2008). Within this diverse community, the genus *Entodinium* is often the most predominant, though its specific abundance varies with host species and diet (Imai et al., 1989; Ito et al., 1994; Henderson et al., 2015; Kišidayová et al., 2021). *Entodinium* species exhibit notable environmental adaptability, efficiently ingesting starch granules and selectively engulfing bacteria. Their daily bacterial consumption can reach up to 24% of the total ruminal bacterial population (Park and Yu, 2023). By hydrolyzing bacterial proteins into amino acids, these ciliates meet their own nutritional demands while simultaneously enhancing cellulose degradation and volatile fatty acid production, thereby substantially contributing to rumen ecosystem homeostasis (Bonhomme, 1990).

To elucidate the detailed physiology and metabolism of rumen ciliates, establishing stable *in vitro* cultures is essential. The pioneering work of Hungate laid the methodological foundation for cultivating rumen ciliates under controlled laboratory conditions (Hungate, 1943). Building on this framework, *in vitro* cultures were subsequently established for several representative species, including *Entodinium simplex*, *Epidinium caudatum*, and *Polyplastron multivesiculatum* (Coleman, 1969; Coleman et al., 1972). In later studies, single-cell transfer techniques emerged as a critical approach for obtaining monocultures of *Ent. caudatum*, and the corresponding culture systems have been progressively optimized and standardized (Dehority, 1998; Park et al., 2017; Park and Yu, 2018a; Park et al., 2021). Although a monoculture of *Ent. furca monolobum* has been employed in previous studies, comprehensive methodological details regarding its establishment and maintenance are lacking (Regensbogenova et al., 2004; Kisidayová et al., 2005). Early studies using axenic cultures of *Ent. caudatum* provided initial evidence that the presence of living bacteria is essential for ciliate survival and growth (Hino and Kametaka, 1977). This bacterial dependence was further confirmed by antibiotic treatment experiments, in which elimination of the associated bacterial community resulted in impaired ciliate viability, whereas normal growth was restored following bacterial supplementation (Park et al., 2017). These observations indicate a fundamental nutritional and metabolic dependence of rumen ciliates on their bacterial partners. Comparable symbiotic relationships in which bacteria promote protozoan growth and proliferation, have been reported across a wide range of protistan taxa. The growth-promoting effect of exogenous *E. coli*, documented in *Ent. caudatum* cultures (Hino and Kametaka, 1977), has also been observed to significantly enhance the growth of *Tetrahymena pyriformis*, *Amoeba proteus*, and *Pseudocohnilembus persalinu* (Jeon, 2006; Siegmund et al., 2013; Xiong et al., 2015). Similarly, *Bacillus licheniformis* enhances the growth of *Balantidium ctenopharyngodoni* by consuming dissolved oxygen and creating favorable anaerobic conditions (Rey et al., 2004; Zhao et al., 2022). Collectively, these findings underscore the critical role of bacteria in protozoan cultivation, functioning as both nutrient sources and ecological modulators.

In our previous work, we established non-axenic monocultures of *Ent. caudatum* WH strain and *Dasytricha ruminantium*, which enabled long-term observation and revealed a profound influence of bacterial community dynamics on ciliate proliferation (Xu et al., 2024a,b). These results further pointed to the essential role of bacteria in sustaining *in vitro* growth. However, despite the accumulating evidences for bacterial dependence, there have been few systematic characterizations of the bacterial communities associated with rumen ciliate monocultures.

Here, we report a detailed protocol that advances the monoculture of *Ent. furca monolobum* and systematic analysis of its associated bacterial community. We isolated and identified bacterial species from both the monoculture supernatant and the ciliate-associated community fractions, evaluating their effects on ciliate proliferation through co-culture experiment. This work not only overcomes the long-standing challenge of maintaining *Ent. furca monolobum in vitro* culture, but also provides a methodological framework for developing defined monoxenic systems. This advancement will facilitate future mechanistic investigations into protozoan-bacterial interactions within the rumen ecosystem.

## 2 Materials and methods

### 2.1 Ethical statement

All animal procedures were performed in accordance with the Regulations for the Administration of Affairs Concerning Experimental Animals and approved by the Institutional Animal Care and Use Committee under approval number JHDXLL2024–018.

### 2.2 *In vitro* monoculture of *Ent. furca monolobum*

#### 2.2.1 Isolation and enrichment

Fresh rumen content was collected from Holstein cows at a farm in Wuhan, Hubei Province, China, following established protocols (Xu et al., 2024b). The rumen content was filtered through four layers of sterile cheesecloth and transported to laboratory immediately at 39°C under anaerobic conditions. The filtrate was transferred into preheated separatory funnels and incubated at 39 °C for 60 min to enrich ciliates. Subsequently, the suspension was sequentially passed through 50-μm and 20-μm nylon meshes to enrich small-sized ciliates. The fraction smaller than 20-μm was inoculated into SP salt solution (Xu et al., 2024b). Morphological observation indicated that approximately 30% of the initial inoculum consisted of *Ent. furca monolobum*.

#### 2.2.2 Purification and monoculture of *Ent. furca monolobum*

A modified SP medium (Xu et al., 2024a) in which the substrate suspension comprised 2% (w/v) whole wheat flour (Xinliang, China) and 2% (w/v) rice starch (Tianpan, China) was used for the culture of *Ent. furca monolobum*. The procedure consisted of two major steps: purification and subsequent establishment of monocultures. Purification was conducted in two stages. First, mixed cultures were maintained for one month under routine feeding and transfer (Xu et al., 2024a). The cultures were then passed through a 10-μm mesh to enrich *Entodinium* species, resulting in preparations in which *Ent. furca monolobum* represented approximately 80% of the population. From this enriched fraction, serial dilution cultures were maintained by transferring 2 mL of culture into 8 mL of fresh medium every three days for one month. The purity of the cultured ciliates was verified by morphological examination and 18S rRNA gene sequencing. For monoculture establishment, 5 mL of the purified suspension was inoculated into 5 mL of modified SP medium and incubated under the same conditions. Ciliate counts were determined microscopically as described previously (Miltko et al., 2015).

#### 2.2.3 Identification of *Ent. furca monolobum*

Morphological identification of *Ent. furca monolobum* cultures was performed with differential interference contrast (DIC) microscopy (Nikon-90i) based on key morphological features such as body size, shape, nucleus, caudal spine structure, and ciliary zone (Williams and Coleman, 1992; Kisidayová et al., 2007; Dehority, 2018; Feng et al., 2020).

For molecular confirmation, genomic DNA was extracted from three replicate samples (50 ciliates each) using the REDExtract-N-Amp™ Tissue PCR Kit (Sigma-Aldrich). The 18S rRNA gene was amplified with ciliate-specific primers (P.324f/P.1747r_2) (Zhang et al., 2015). The PCR conditions were as follows: 94 °C for 3 min; 32 cycles of 94 °C for 30 s, 56 °C for 1 min, 72 °C for 2 min; final extension 72 °C for 7 min. PCR products were sequenced (Sangon Biotech, Wuhan, China) and aligned against GenBank sequences for species identification.

Phylogenetic analysis was performed in PhyloSuite v1.2.3 (Zhang et al., 2020) using 27 rumen ciliate 18S rRNA sequences and three outgroups. Alignments were generated with MAFFT v7.505 (Katoh and Standley, 2013) and refined using Gblocks v0.91b (Talavera and Castresana, 2007). The GTR + F + I + G4 model was selected with ModelFinder (Kalyaanamoorthy et al., 2017). Bayesian inference (BI) analysis was performed with MrBayes v3.2.7a for 2,000,000 generations (sampling every 1,000 generations) (Ronquist et al., 2012). Maximum likelihood (ML) analysis was conducted using IQ-TREE v2.2.0 (Nguyen et al., 2015) with 1,000 bootstrap replicates. Phylogenetic trees were visualized in iTOL.^1^

### 2.3 Isolation and identification of growth-promoting bacteria

#### 2.3.1 Bacterial media and culture conditions

Since CMC-Na agar medium is effective for isolating functional rumen bacteria (Sari et al., 2017; Rawway et al., 2018) and nutrient agar medium serves as a common bacterial culture, isolates from the monoculture supernatant and the ciliate-associated community were maintained on the both medium. It should be noted that this culture-dependent protocol selectively recovers only a subset of cultivable bacteria and therefore does not comprehensively represent the original bacterial community. The media were prepared as previously described (Li et al., 2009; Kumari and Naraian, 2021). Given that the bacterial community in long-term laboratory monoculture conditions comprises taxa tolerant to micro-oxic conditions, isolates were performed under both aerobic and anaerobic conditions at 39 °C. Purified bacterial isolates were preserved in LB broth (Wang et al., 2023) and propagated at 39 °C with shaking at 180 rpm for 8 h.

#### 2.3.2 Isolation and purification of bacteria from the monoculture supernatant

Under both aerobic and anaerobic conditions, 100 μL of monoculture supernatant was serially diluted with sterile saline solution from 10^−1^ to 10^−3^. An aliquot of 100 μL from each dilution was spread onto nutrient agar and CMC-Na agar plates using a sterile spreader, and then incubated at 39 °C for 12–24 h under the corresponding conditions. Meanwhile, control plates were prepared by spreading an equivalent volume of the sterile saline solution. Distinct single colonies were selected and sub-cultured using the streak plate method until pure isolates were obtained. Purified colonies were inoculated into LB broth and grown at 39 °C with shaking at 180 rpm for 8 h. Gram staining was used for preliminary characterization.

#### 2.3.3 Isolation and purification of ciliate-associated bacteria

Ciliate-associated bacteria were obtained according to the methods described by Gutierrez (1958). Briefly, 50 ciliates were collected and washed on an 8-well glass concavity slide pre-filled with sterile SP salt solution using a sterile micropipette, then transferred to a sterile glass slide for lysis by crushing with a second slide. Several drops of sterile saline solution were then added to the crushed ciliates to generate a suspension. This suspension was diluted (10^−1^–10^−2^) and plated as above under aerobic/anaerobic conditions. As a negative control, an equivalent volume of sterile saline solution was spread on control plates. Colonies were purified, Gram-stained, and maintained in LB broth at 39 °C.

#### 2.3.4 Molecular characterization of isolated strains and phylogenetic analysis

DNA was extracted using the REDExtract-N-Amp™ Tissue PCR Kit. The 16S rRNA gene was amplified with universal primers 27F/1492R (Dos Santos et al., 2019). Thermal cycling was performed under the following conditions: 95 °C for 5 min; 30 cycles of 94 °C for 30 s, 55 °C for 30 s, 72 °C for 90 s; final extension 72 °C for 10 min. Amplicons were sequenced (Sangon Biotech) and aligned with GenBank for taxonomic identification. Phylogenetic analysis followed the same pipeline as above.

#### 2.3.5 Screening of growth-promoting bacteria

##### 2.3.5.1 Assessment of bacterial growth-promoting effects on *Ent. furca monolobum*

The bacterial strains common to both the monoculture supernatant and ciliate-associated community were selected for co-culture with *Ent. furca monolobum*, as they represent potential symbiotic bacteria and were presumed to possess growth-promoting effects. Accordingly, bacterial suspensions (10^7^ CFU/mL) were supplemented daily at 0.3% (v/v) to *Ent. furca monolobum* monocultures to establish co-culture systems for evaluating their growth-promoting effects. Controls received a daily supplement of modified SP medium at an equivalent volume.

Fresh rumen fluid was first centrifuged at 3,000 × g for 3 min to remove ciliates, and the supernatant was then subjected to centrifugation at 10,000 × g for 10 min to prepare RF group. After co-culture, ciliates from monocultures without bacterial supplementation were collected, washed five times in sterile SP salt solution by centrifugation at 500 × g for 3 min (supernatant removed after each step), and the resulting pellet was designated as Cm group. To obtain control supernatant bacteria, the supernatant was filtered through a 2.6-μm nylon mesh to remove ciliates, followed by centrifugation at 10,000 × g for 10 min to collect bacteria and labeled as Cs group. Co-cultures supplemented with *E. coli* or *E. fergusonii* were processed similarly and designated as Em/Es and Fm/Fs groups, respectively. All samples were stored at −80 °C prior to DNA extraction and amplicon sequencing. During the co-culture experiment, the growth of *Ent. furca monolobum* was monitored by daily cell counting, and growth curves were plotted from these data.

##### 2.3.5.2 Microbiota analysis using amplicon sequencing

Total genomic DNA was extracted using the cetyltrimethylammonium bromide (CTAB) method (Villegas-Rivera et al., 2013). The V4 region of the 16S rRNA gene was amplified with primers (515F: 5′-GTGCCAGCMGCCGCGGTAA-3′ and 806R: 5′-GGACTACHVGGGTWTCTAAT-3′) (Park and Yu, 2018b). The PCR conditions were as follows: 98°C for 1 min; 30 cycles of 98°C for 10 s, 50°C for 30 s, 72°C for 30 s; final extension 72 °C for 5 min. PCR products were purified and used to construct libraries (NEBNext Ultra II DNA Library Prep Kit), which were sequenced on Illumina NovaSeq 6,000 (PE250).

Raw sequences were processed with the DADA2 pipeline (Callahan et al., 2016) for quality filtering, denoising, and paired-end reads merging to generate amplicon sequence variants (ASVs). Taxonomic annotation of the ASVs was performed using the QIIME 2’s q2-feature-classifier plugin, with the SILVA v138.1 database as the reference (Quast et al., 2013; Bokulich et al., 2018). Based on the taxonomic annotation results, we generated taxonomic abundance profiles across defined phylogenetic level.

### 2.4 Statistical analysis

All experiments were conducted with three replicates. Growth data were analyzed using a two-way repeated measures ANOVA with Greenhouse–Geisser correction and Tukey’s test. Statistical significance was set at *p* < 0.05. Data visualization was performed using Origin 2024 (OriginLab Corporation, Northampton, MA, USA).

## 3 Results

### 3.1 *In vitro* monoculture of *Ent. furca monolobum*

**Figure 1:**
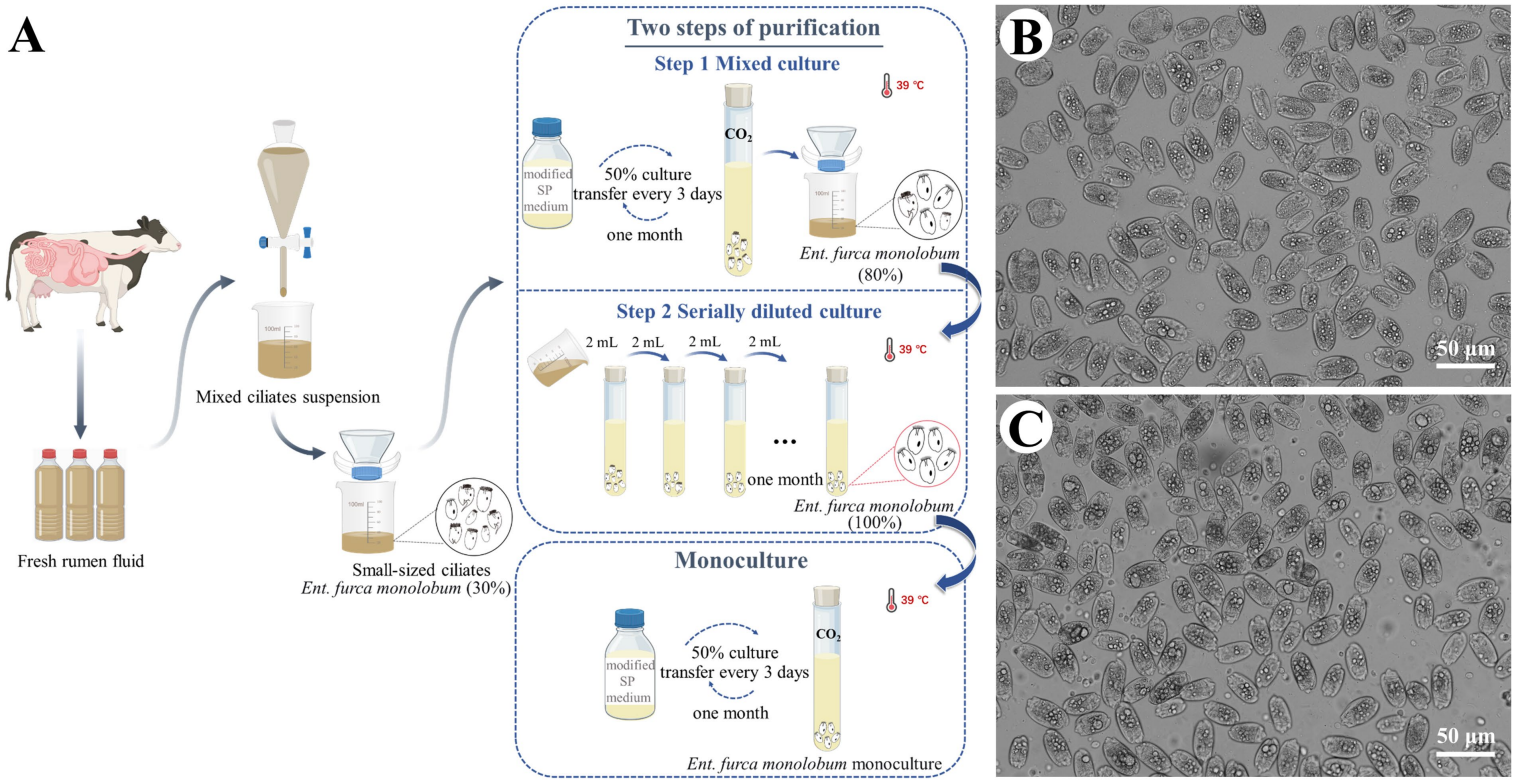
*In vitro* culture establishment and the proportion of *Entodinium furca monolobum*. **(A)** Diagram illustrating the isolation and two-step purification procedures used to establish the monoculture. **(B, C)** Relative abundance of *Ent. furca monolobum* before **(B)** and after **(C)** purification. Image created with BioRender web.

Based on our previous experience with monoculture of *Ent. caudatum* WH strain and *D. ruminantium*, we adopted a two-step strategy involving mixed and serially diluted cultures to purify *Ent. furca monolobum in vitro* (Figure 1A). An enriched inoculum was first maintained in mixed culture for one month, yielding a population in which *Ent. furca monolobum* accounted for approximately 80% (Figure 1B). Subsequently, serial dilution purification was conducted for another month, resulting in a culture comprising 100% *Ent. furca monolobum* (Figure 1C).

**Figure 2:**
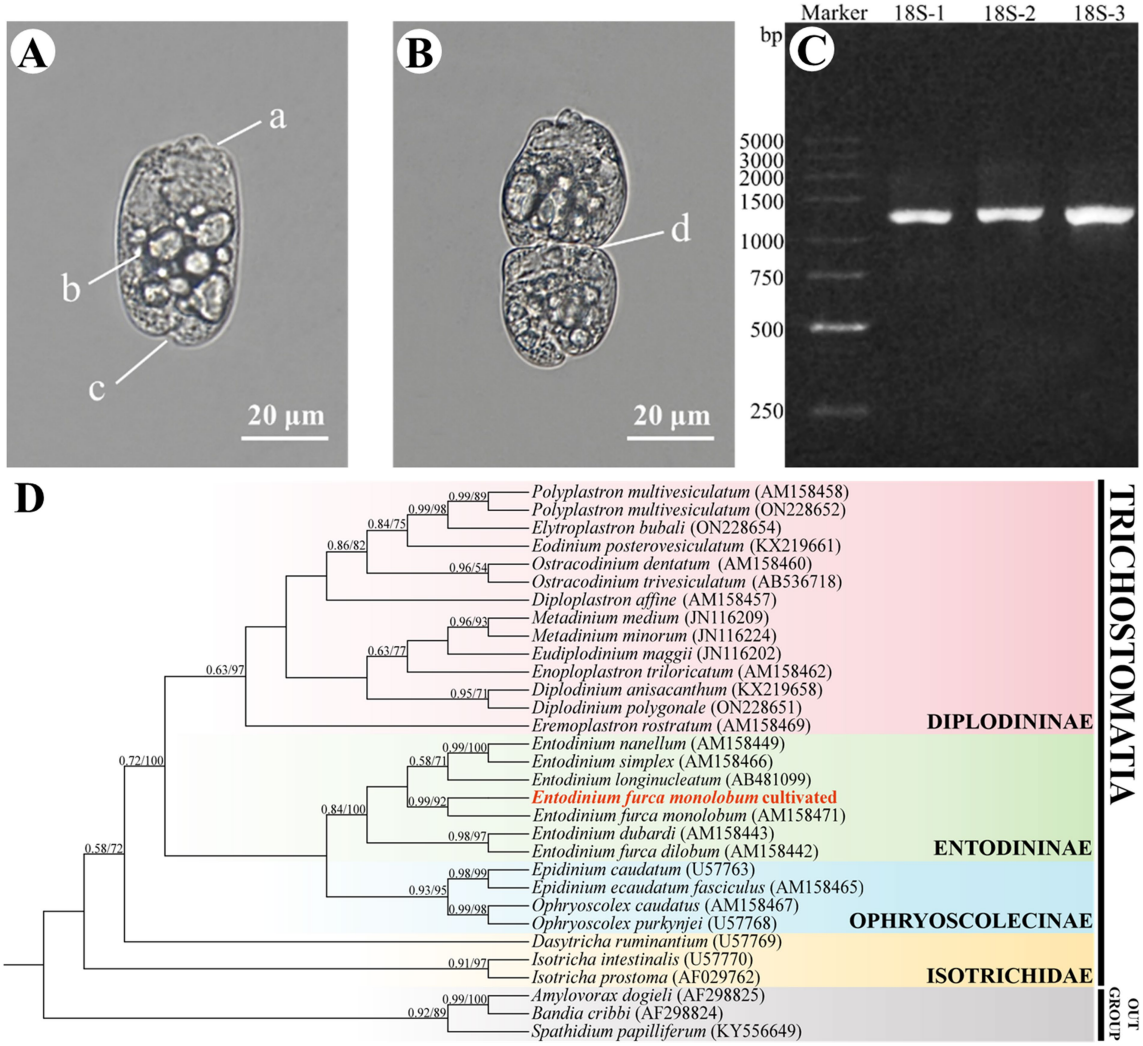
Morphological and molecular identification of *Entodinium furca monolobum* from *in vitro* monoculture. **(A, B)** Morphological features of *Ent. furca monolobum.* **(A)** Representative cell from the *Ent. furca monolobum* monoculture. **(B)** Dividing cell showing the formation of ciliary anlage. Key structures are indicated: a-cilia; b-food vacuole; c-cytoproct; d-ciliary anlage of dividing *Ent. furca monolobum*. **(C)** PCR amplification of 18S rRNA gene from monocultured *Ent. furca monolobum.* **(D)** Phylogenetic analysis of rumen ciliates based on 18S rRNA gene sequences. Numbers at nodes indicate Bayesian posterior probability (left) and maximum-likelihood bootstrap values (right), only values >0.5/50 are shown. Newly obtained sequence is highlighted in bold red.

Morphological observations confirmed that the culture was exclusively composed of *Ent. furca monolobum*. The cells exhibited a small and elongated-oval morphology, measuring 25–40 μm in length and 20–30 μm in width, with no skeletal plates observed. The anterior end was truncate, and caudal spines were absent. A ciliary zone was present adjacent to the vestibulum (Figure 2A). The cilia were extended externally during feeding and locomotion but retracted into the cell upon exposure to unfavorable environmental conditions. Multiple food vacuoles were observed within the cytoplasm. These morphological traits are consistent with the descriptions by Dehority (2018), indicating the monoculture is *Ent. furca monolobum*. Moreover, actively dividing cells were observed in monoculture (Figure 2B). During division, a new ciliary apparatus primordium formed at the mid-body, eventually separating to produce two individual cells, with the primordium developing into the oral ciliary zone of the daughter cell. These results demonstrated that *Ent. furca monolobum* can be stably maintained and proliferated effectively in the modified SP medium.

The 18S rRNA gene of the monoculture was successfully amplified and sequenced (Figure 2C). BLAST analysis indicated that the obtained sequence (Accession number: PX735707) shared the highest identity with the 18S rRNA gene of *Ent. furca monolobum* (Accession number: AM158471.1), exhibiting 99.80% sequence identity and an *E*-value of 0. The topologies of the BI and ML trees are congruent. Figure 2D shows the topology of the BI tree, with support values derived from both the BI and ML analyses indicated on the branches. The 18S rRNA sequence of the monocultured ciliates clustered as a sister group to the reported *Ent. furca monolobum* sequence from GenBank with strong support (0.99 BI/92% ML). Therefore, the monocultured ciliates were *Ent. furca monolobum*.

### 3.2 Isolation and identification of associated bacteria of *Ent. furca monolobum* monoculture

#### 3.2.1 Isolation and purification of bacteria from the monoculture supernatant and ciliate-associated community

From the monoculture supernatant, 23 bacterial strains were obtained under different culture conditions (Supplementary Table 1). Under aerobic conditions, six morphologically distinct colonies (S1–S6, accession numbers PV981802-981807) were obtained on nutrient agar medium, and seven colonies (S7–S13, accession numbers PV981808-981814) were obtained on CMC-Na agar medium. Anaerobic cultivation yielded 10 distinct colonies (S14–S23, accession numbers PV981815-981824) on nutrient agar medium.

**Figure 3:**
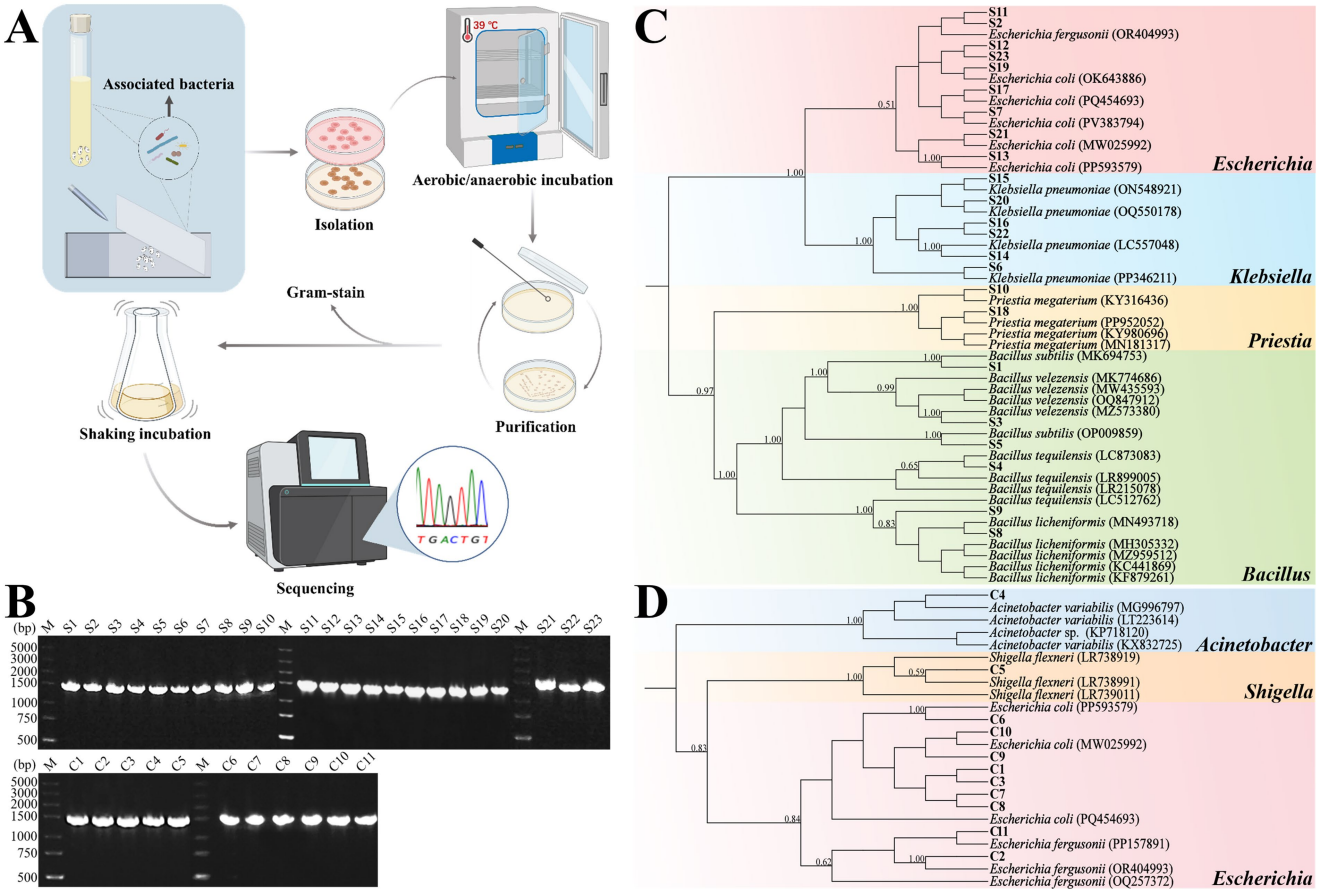
Isolation, purification, and phylogenetic identification of associated bacteria from *Entodinium furca monolobum* monoculture. **(A)** Schematic workflow illustrating the isolation and identification procedures of associated bacteria. **(B)** PCR amplification of the 16S rRNA gene from bacterial isolates obtained from the monoculture supernatant (S1–S23) and the ciliate-associated community (C1–C11); M denotes marker. **(C, D)** Phylogenetic trees of bacterial isolates derived from the monoculture supernatant **(C)** and the ciliate-associated community **(D)**. Numbers at nodes indicate Bayesian posterior probability values; only values >0.5 are shown. Newly obtained sequences are highlighted in bold. Image created with BioRender web.

From the ciliate-associated community, 11 distinct strains were isolated (Supplementary Table 2). Aerobic cultivation yielded four colonies (C1–C4, accession numbers PV981825-981828) on nutrient agar medium and two colonies (C5–C6, accession numbers PV981829-981830) on CMC-Na agar medium. Anaerobic cultivation resulted in three colonies (C7–C9, accession numbers PV981831-981833) on nutrient agar medium and two colonies (C10–C11, accession numbers PV981834-981835) on CMC-Na agar medium. In contrast, no colonies were observed on any of the control plates. The experimental workflow was shown in Figure 3A.

#### 3.2.2 Identification of isolated bacterial strains

Genomic DNA was extracted from 34 bacterial isolates, including 23 strains from the monoculture supernatant and 11 strains from the ciliate-associated community. The PCR amplifications of 16S rRNA gene yielded products of approximately 1,400 bp (Figure 3B).

The obtained 16S rRNA gene sequences were analyzed by BLAST against the NCBI database to validate species-level assignments (Supplementary Tables 1, 2), and phylogenetic trees were further evaluated the taxonomic affiliations. The 23 isolates from the monoculture supernatant were classified into eight species: *E. coli*, *E. fergusonii*, *Klebsiella pneumoniae*, *Priestia megaterium*, *B. licheniformis*, *B. subtilis*, *B. tequilensis*, and *B. velezensis* (Figure 3C). The 11 isolates from the ciliate-associated community were assigned to four species: *E. coli*, *E. fergusonii*, *Shigella flexneri*, and *Acinetobacter* *variabilis* (Figure 3D).

#### 3.2.3 Screening of growth-promoting bacteria

##### 3.2.3.1 Effects of bacteria strains on the proliferation of *Ent. furca monolobum*

**Figure 4:**
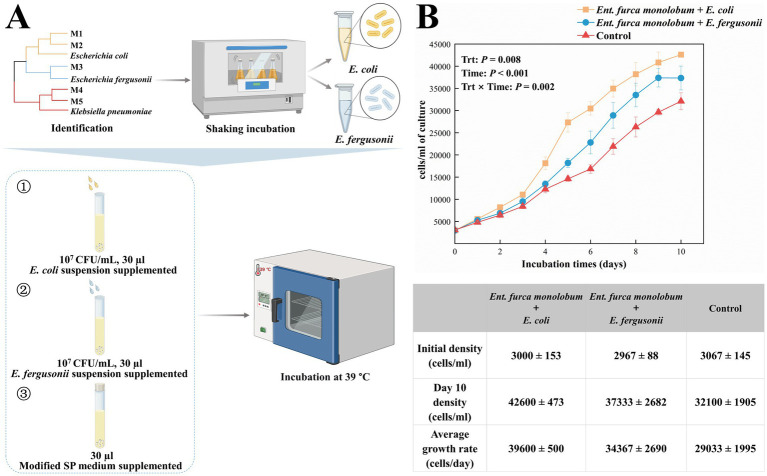
Screening of growth-promoting bacteria and their effects on the population growth of *Entodinium furca monolobum*. **(A)** Graphical representation of the experimental process. Monocultures were initiated at an initial density of 3 × 10^3^ cells mL^−1^ and supplemented daily with 30 μL of bacterial suspensions (10^7^ CFU/mL). Monocultures supplemented with an equivalent volume of modified SP medium were used as controls. **(B)** Growth curves of *Ent. furca monolobum* under three culture conditions. Growth parameters include the initial density, population density on day 10 (Day 10 density), and the average growth rate. Data are expressed as mean ± SEM from three replicates of each group. Trt, treatment. Statistically significant differences among the three groups are indicated (*p* < 0.05). Image created with BioRender web.

Comparative analysis of the bacterial isolates from the monoculture supernatant and the ciliate-associated community, we found that *E. coli* and *E. fergusonii* were species common to both sources. To assess their growth-promoting potential, monocultures of *Ent. furca monolobum* were separately supplemented with each bacterial suspension (10^7^ CFU/mL, 30 μL). Monocultures supplemented with an equal volume of modified SP medium served as controls (Figure 4A). Monocultured *Ent. furca monolobum* were inoculated at an initial density of 3,000 cells/mL and received a daily supplementation of 30 μL of the respective bacterial suspension. Across the 10-day cultivation, bacterial supplementation increased the proliferation of *Ent. furca monolobum* (Figure 4B). Statistical analysis revealed significant main effects of treatment (*p* = 0.008) and time (*p* < 0.001), as well as a significant treatment × time interaction (*p* = 0.002), indicating that the growth trajectories differed meaningfully among the groups. Specifically, on Day 10, significant differences in cell density among treatments were confirmed by Tukey’s *post hoc* test. The *E. coli* treatment reached the highest density (42,600 ± 473 cells/mL), which was significantly greater than both the control (32,100 ± 1,905 cells/mL; *p* < 0.0001) and the *E. fergusonii* group (37,333 ± 2,682 cells/mL; *p* < 0.05). The *E. fergusonii* treatment also showed a significantly higher cell density than the control (*p* < 0.05).

##### 3.2.3.2 Bacterial community diversity and structure

**Figure 5:**
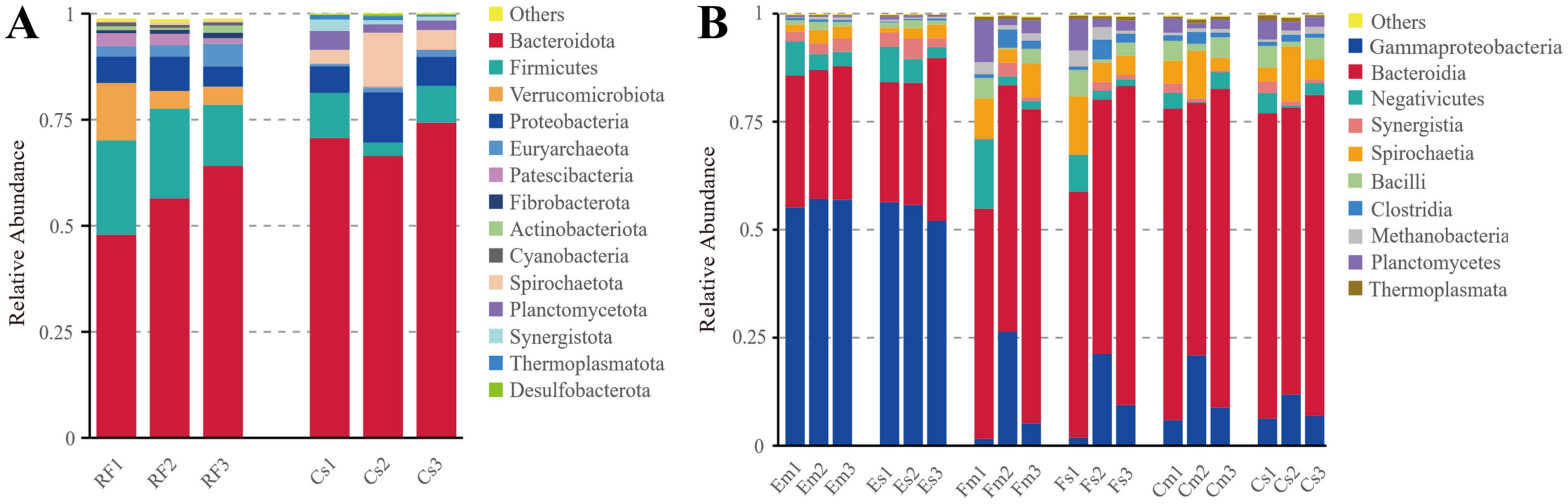
Relative abundance of dominant bacterial taxa based on 16S rRNA amplicon sequencing. **(A)** Relative abundance of the 10 most abundant bacterial phyla detected in protozoa-free rumen fluid and the monoculture supernatant. **(B)** Relative abundance of the 10 most abundant bacterial classes from the monoculture supernatant and the ciliate-associated bacterial community under different co-culture treatments. RF: Bacteria from protozoa-free rumen fluid; Cs: Bacteria from the monoculture supernatant without bacterial addition; Cm: Bacteria from the ciliate-associated community without bacterial addition; Es: Bacteria from the monoculture supernatant with *Escherichia coli* addition; Em: Bacteria from the ciliate-associated community with *E. coli* addition; Fs: Bacteria from the monoculture supernatant with *Escherichia fergusonii* addition; Fm: Bacteria from the ciliate-associated community with *E. fergusonii* addition.

A comparative analysis of the bacterial community composition between protozoa-free rumen fluid and the monoculture supernatant were conducted using high-throughput 16S rRNA gene amplicon sequencing. At the phylum level, the microbiota of protozoa-free rumen fluid was predominantly composed of Bacteroidetes (56.15%) and Firmicutes (19.26%). However, the bacterial communities of the monoculture supernatant were largely dominated by Bacteroidetes (70.48%), followed by Proteobacteria (7.49%) (Figure 5A).

Amplicon sequencing of 18 samples from six experimental groups (Em, Es, Fm, Fs, Cm, Cs) yielded 1,936,797 raw sequences. After quality filtering and chimera removal, 1,849,066 effective sequences were retained, with an average length of 252.89 ± 0.065 bp. Denoising performed with DADA2 under 100% identity clustering yielded 898 ASVs. The Cs group exhibited the highest ASV richness (409 ASVs), while the Em group showed the lowest (273 ASVs).

The bacterial community composition of each treatment group at the class level was shown in Figure 5B. The Em and Es groups displayed similar profiles, comprising 21 and 20 bacterial classes, respectively. In both Em and Es groups, Gammaproteobacteria was the most abundant class (56.39 and 54.73%), followed by Bacteroidia (30.41 and 31.21%) and Negativicutes (5.00 and 5.41%). The Fm and Fs groups contained 21 and 20 classes, with Bacteroidia representing the largest taxa (61.03 and 63.20%), followed by lower abundances of Gammaproteobacteria (11.05 and 10.82%) and Spirochaetia (6.72 and 7.49%). Both Cm and Cs groups contained 20 classes, with Bacteroidia remaining the most dominant one (68.10 and 70.48%), followed by Gammaproteobacteria (11.89 and 8.32%) and Spirochaetia (6.36 and 6.86%).

## 4 Discussion

*Entodinium* is the most prevalent genus of rumen ciliates and exhibits the strongest capacity for bacterial ingestion due to its high abundance (Belanche et al., 2012). In this study, we successfully established a monoculture of *Ent. furca monolobum*, providing a robust model for exploring microbial physiology and ciliate–bacteria interactions in the rumen. Referring to the method of monocultured *Ent. caudatum* developed in our lab (Xu et al., 2024a), we optimized the purification process by repeated filtration through a 10 μm nylon mesh, and improved the culture substrate by replacing wheat flour and alfalfa meal with whole wheat flour and rice starch. The latter provide more abundant nutrients (e.g., vitamins and minerals), thereby supporting stable ciliate growth. Notably, the bacterial community in the monoculture supernatant differed markedly from that in protozoa-free rumen fluid. While the *in vitro* system is intended to mimic the rumen environment, it is substantially less complex than the native ecosystem, favoring the proliferation of bacterial taxa capable of rapid adaptation.

Rumen ciliates exhibit considerable interspecific variability in their dependence on bacteria and feeding preferences (Park and Yu, 2018b). Although bacterial proteins generally promote ciliate proliferation, different ciliate species display distinct nutritional strategies. For example, smaller *Entodinium* species such as *Ent. caudatum* and *Ent. simplex* rely heavily on bacteria for nitrogen and energy supply, thereby increasing bacterial consumption rates (Bonhomme, 1990). In contrast, *Epi. caudatum* consumes fewer bacteria but utilizes free amino acids more efficiently (Bonhomme, 1990). Other genera such as *Eudiplodinium* and *Isotricha* can exploit both bacterial and environmental amino acids, whereas *P. multivesiculatum* primarily depends on free amino acids to meet its nitrogen demands (Bonhomme, 1990). Previous studies have reported a prevalence of Proteobacteria in association with certain rumen ciliates (Park and Yu, 2018b). Our finding that bacteria from this phylum (specifically *Escherichia* spp.) promote ciliate growth raises the possibility that such associations may be functionally significant, contributing to the stability of the ciliate population and, by extension, the rumen microbial ecosystem.

Ciliate-associated bacteria play a crucial role in sustaining ciliate growth, acting as essential nutrient providers as well as ecological modulators. Previous studies combining electron microscopy and bioinformatics revealed diverse bacteria within culture supernatants and associated with ciliates. It is now well-established that bacterial supplementation has greatly facilitated the establishment of *in vitro* cultures for a wide range of ciliate species (Siegmund et al., 2013; Xiong et al., 2015; Zhao et al., 2022). Beyond serving as prey, bacteria provide amino acids, nucleic acids, vitamins, and even create favorable anaerobic conditions for ciliate survival (Bonhomme, 1990). Guided by these insights, we investigated whether isolates from the monoculture supernatant and the ciliate-associated community could promote the growth of *Ent. furca monolobum*. The isolates common to both sources may suggest the establishment of a close and functionally cooperative association with the ciliate. And our findings revealed that both *E. coli* and *E. fergusonii* significantly enhanced ciliate proliferation, with *E. coli* exerting a stronger effect. The superior promoting effect of *E. coli* may be attributed to its enhanced capacity to produce and secrete growth-stimulating metabolites that positively modulate ciliate metabolic pathways.

To our knowledge, this is the first report identifying bacterial strains that promote the *in vitro* growth of *Ent. furca monolobum.* The culture methods established here provide a valuable reference for optimizing the cultivation of other anaerobic rumen ciliates. However, it should be acknowledged that culture-based isolation is selective. The media and incubation conditions used here are unlikely to recover many fastidious and strictly anaerobic bacterial taxa. Therefore, the isolates obtained represent only a readily cultivable fraction of the community and should not be interpreted as representative of the *in situ* rumen microbiota. In light of this constraint, our strategy intentionally prioritized isolates that could persist over long-term laboratory culture conditions and maintain interactions with the ciliates. The *Escherichia* spp. isolates we obtained enhanced ciliate growth, indicating their functional relevance. Moreover, this co-culture system offers a tractable experimental model for dissecting ciliate–bacteria mutualism, including mechanisms such as bacterial evasion of digestion and secretion of growth factors (Siegmund et al., 2013; Zhang et al., 2024). In future studies, development of a monoxenic culture system of *Ent. furca monolobum* using a defined medium supplemented with a single bacterial partner would allow precise analysis of their interactions. Ultimately, these targeted co-culture strategies provide a theoretical foundation for establishing efficient and stable *in vitro* cultivation systems for rumen ciliates and offer new opportunities for manipulating rumen microbial ecosystems through key microbial partners.

## 5 Conclusion

This study reports the successful establishment of a stable monoculture of *Ent. furca monolobum* using a modified SP medium, thereby overcoming the long-standing limitation of its reliance on mixed ciliate cultures and the lack of a defined cultivation protocol. Through isolation and identification of bacteria from both the monoculture supernatant and the ciliate-associated community, *E. coli* and *E. fergusonii* were identified as key growth-promoting species. Co-culture experiment confirmed their ability to enhance ciliate proliferation, with microbial community analysis further revealing a shift toward Gammaproteobacteria dominance in cultures supplemented with *E. coli*. These findings provide a methodological breakthrough for establishing monoxenic cultures of *Ent. furca monolobum*, offering a valuable model to investigate protozoan physiology and laying the foundation for developing *in vitro* cultivation strategies for other anaerobic ciliates.

## Data Availability

The datasets presented in this study have been deposited in GenBank (https://www.ncbi.nlm.nih.gov/genbank/) under accession numbers PX735707 and PV981802-PV981835.

## Footnotes

1
http://itol.embl.de/
