# Supplementary material for: Establishment of a stable monoculture system for Entodinium furca monolobum and isolation of Escherichia spp. as growth-promoting bacteria
Source: Front Microbiol. 2026 Feb 12;17:1741192. doi: 10.3389/fmicb.2026.1741192 (PMC12935935; doi:10.3389/fmicb.2026.1741192)
Supplement: Supplementary file 1 [file Table_1.docx]

**Supplementary Table 1. Identification and characterization of bacterial isolates from the monoculture supernatant (pigmentation, shape, gram staining and 16S rRNA gene-based identification).**

| **Strain** | **Morphological and biochemical**  **characters** | | |  | **Identification** | |
| --- | --- | --- | --- | --- | --- | --- |
|  | **Colony**  **color** | **Colony shape** | **Gram stain** |  | **Closest**  **organism** | **Identity (%)** |
| S1 | Cream white | Irregular | + |  | *Bacillus tequilensis* | 99.93% |
| S2 | Cream yellow | Circular | - |  | *Escherichia fergusonii* | 99.72% |
| S3 | Cream white | Irregular | + |  | *Bacillus velezensis* | 99.79% |
| S4 | Cream white | Irregular | + |  | *Bacillus tequilensis* | 99.79% |
| S5 | Cream white | Irregular | + |  | *Bacillus subtilis* | 99.79% |
| S6 | Cream yellow | Circular | - |  | *Klebsiella* sp. | 98.09% |
| S7 | Cream white | Circular | - |  | *Escherichia coli* | 99.79% |
| S8 | Cream white | Circular | + |  | *Bacillus licheniformis* | 99.93% |
| S9 | Cream white | Circular | + |  | *Bacillus licheniformis* | 99.86% |
| S10 | Cream yellow | Circular | + |  | *Priestia megaterium* | 99.93% |
| S11 | Cream white | Circular | - |  | *Escherichia fergusonii* | 99.65% |
| S12 | Cream white | Circular | - |  | *Escherichia coli* | 99.86% |
| S13 | Cream white | Irregular | - |  | *Escherichia coli* | 99.79% |
| S14 | Cream yellow | Circular | - |  | *Klebsiella pneumoniae* | 99.86% |
| S15 | Cream yellow | Circular | - |  | *Klebsiella pneumoniae* | 99.86% |
| S16 | Cream yellow | Circular | - |  | *Klebsiella pneumoniae* | 99.86% |
| S17 | Cream white | Circular | - |  | *Escherichia coli* | 99.93% |
| S18 | Cream yellow | Circular | + |  | *Priestia* sp. | 99.86% |
| S19 | Cream white | Circular | - |  | *Escherichia coli* | 99.79% |
| S20 | Cream white | Circular | - |  | *Klebsiella pneumoniae* | 99.93% |
| S21 | Cream white | Circular | - |  | *Escherichia coli* | 99.72% |
| S22 | Cream white | Circular | - |  | *Klebsiella pneumoniae* | 99.86% |
| S23 | Cream yellow | Circular | - |  | *Escherichia coli* | 99.79% |

Note: +, positive; -, negative.
