# Supplementary material for: Establishment of a stable monoculture system for Entodinium furca monolobum and isolation of Escherichia spp. as growth-promoting bacteria
Source: Front Microbiol. 2026 Feb 12;17:1741192. doi: 10.3389/fmicb.2026.1741192 (PMC12935935; doi:10.3389/fmicb.2026.1741192)
Supplement: Supplementary file 2 [file Table_2.docx]

**Supplementary Table 2. Identification and characterization of bacterial isolates from the ciliate-associated community (pigmentation, shape, gram staining and 16S rRNA gene-based identification).**

| **Strain** | **Morphological and biochemical**  **characters** | | |  | **Identification** | |
| --- | --- | --- | --- | --- | --- | --- |
|  | **Colony colour** | **Colony shape** | **Gram stain** |  | **Closest**  **organism** | **Identity（%）** |
| C1 | Cream white | Circular | - |  | *Escherichia coli* | 99.86% |
| C2 | Cream white | Circular | - |  | *Escherichia coli* | 99.72% |
| C3 | Cream white | Circular | - |  | *Escherichia coli* | 99.58% |
| C4 | Cream yellow | Circular | - |  | *Acinetobacter variabilis* | 99.86% |
| C5 | Cream white | Circular | - |  | *Shigella flexneri* | 99.72% |
| C6 | Cream yellow | Circular | - |  | *Escherichia coli* | 99.72% |
| C7 | Cream white | Circular | - |  | *Escherichia coli* | 99.86% |
| C8 | Cream yellow | Circular | - |  | *Escherichia coli* | 99.86% |
| C9 | Cream white | Circular | - |  | *Escherichia coli* | 99.79% |
| C10 | Cream white | Circular | - |  | *Escherichia coli* | 99.58% |
| C11 | Cream yellow | Circular | - |  | *Escherichia fergusonii* | 99.86% |

Note: +, positive; -, negative.
